# Differences in Pathological Composition Among Large Artery Occlusion Cerebral Thrombi, Valvular Heart Disease Atrial Thrombi and Carotid Endarterectomy Plaques

**DOI:** 10.3389/fneur.2020.00811

**Published:** 2020-08-07

**Authors:** Yu Liao, Min Guan, Dan Liang, Yingying Shi, Jialin Liu, Xiuli Zeng, Shengming Huang, Xiaomei Xie, Dingxin Yuan, Hongyu Qiao, Li'an Huang

**Affiliations:** ^1^Department of Neurology, Clinical Neuroscience Institute, The First Affiliated Hospital, Jinan University, Guangzhou, China; ^2^Department of Pathology, The First Affiliated Hospital, Jinan University, Guangzhou, China; ^3^Department of Neurology, Beijiao Hospital, Foshan, China

**Keywords:** stroke, mechanical thrombectomy, thrombus, red blood cells, white blood cells, fibrin, platelets

## Abstract

**Background and Purpose:** Acute ischemic stroke (AIS) with large artery occlusion (LAO) may lead to severe disability or death if not promptly treated. To determine the source of cerebral artery occlusion thrombosis, we studied the pathological components of cerebral artery thrombosis with different etiological classifications to guide clinical formulation of preventive treatment.

**Materials and Methods:** Eighty-eight thrombi from AIS patients with LAO, 12 atrial thrombi from patients with valvular heart disease (VHD), and 11 plaques obtained by carotid endarterectomy (CEA) from patients with carotid artery stenosis were included in this retrospective study. The hematoxylin and eosin–stained specimens were quantitatively analyzed for erythrocytes, white blood cells (WBCs) and fibrin; platelets were shown by immunohistochemistry for CD31.

**Results:** The thrombi of VHD showed the highest percentage of fibrin, followed by those of cardioembolism (CE) and stroke of undetermined etiology (SUE), and these values were higher than those of the other groups. Plaques obtained by CEA showed the highest erythrocyte number, followed by the large artery atherosclerosis (LAA) thrombi, and showed significantly noticeable differences between other stroke subtypes. The proportions of fibrin and erythrocytes in the thrombi of CE and SUE were most similar to those in the thrombi of VHD, and the LAA thrombi were the closest to those obtained by CEA. CE thrombi and CEA plaques had a higher percentage of WBCs than thrombi of other stroke thrombus subtypes and VHD.

**Conclusions:** CE and most cryptogenic thrombi may originate from the heart, and the formation of carotid atherosclerotic plaques may be related to atherosclerotic cerebral embolism. Inflammation may be involved in their formation.

## Introduction

Acute cerebral large artery occlusion (LAO) may lead to severe disability or even death if the patient does not have access to prompt treatment. In recent years, the benefit of endovascular therapy has been demonstrated in patients. Embolic clots of the cerebral large arteries may come from deciduous cardiac valvular thrombi or intracranial/extracranial large artery plaques. Thus, it is important to identify the etiology of LAO in clinical treatment (1). Although the TOAST classification is currently simple and easy to use and advances imaging and diagnostic methods, identifying a clear stroke etiology remains challenging for a certain percentage of stroke patients, especially for patients with atrial fibrillation and intracranial large vascular stenosis or paroxysmal atrial fibrillation, and the etiological mechanisms are difficult to obtain from clinical data (2–4). The study of thrombus properties and components provides good guidance in the selection of recanalization treatment. Previously, assessment of thrombus length by imaging has been used as an independent predictor of the success of vein recanalization, but no histopathological evidence has been verified.

Staessens et al. found that all thrombi were composed of platelet-rich regions and erythrocyte-rich regions by analyzing the composition and internal structure of AIS thrombi retrieved from endovascular therapy; platelet-rich areas are composed of fibrin, von Willebrand's factor (vWF) and platelets. They also suggested that platelet-rich thrombi based on vWF and DNA as well as dense fibrin were the main reason for the failure of intravenous thrombolysis, and the histological characteristics of platelets, vWF and fibrin networks in thrombi were observed by fluorescence microscopy (5). Recent studies have demonstrated that arterial thrombi are mainly composed of the following three components: fibrin/platelet aggregation, red blood cells and white blood cells (WBCs) (6, 7). However, the source of thrombi in patients with AIS is more complex than that of thrombi in the coronary arteries, mainly from the cardiac or large arteries. CE thrombi have higher proportions of fibrin, fewer red blood cells, and more WBCs than non-cardioembolic thrombi. The thrombus histology of cryptogenic strokes and CE strokes showed a strong overlap (8–10). However, Kim et al. observed that CE thrombi had higher proportions of red blood cells than LAA thrombi, and CE thrombi had fewer fibrin, platelets, and WBCs, although the differences were not statistically significant. There were also no statistically significant differences in the proportion of thrombi components between SUE, CE, and LAA causes (11). No consistent results have been reported regarding the association between thrombi compositions and stroke subtypes, and these studies focused on only thrombi with AIS, with relatively limited results. Based on this inconsistency, this is the first study to perform pathologic analysis of atrial thrombi, carotid atherosclerotic plaques, and LAO thrombi to compare the distribution of fibrin/platelets, red blood cells, and WBCs. We attempted to clarify the pathogenesis and origin of stroke thrombi by analyzing differences in thrombi composition.

## Methods

### Patient Selection

(1) This retrospective study comprised 263 Chinese patients with LAO from January 2017 to March 2019. This study was conducted according to the recommendations of guidelines from the Institutional Review Board of the First Affiliated Hospital of Jinan University. All protocols and procedures of our research were carried out in conformity to the Helsinki Declaration. All patients who were treated in our hospital signed informed consent for medical research of their images and specimens.

The inclusion criteria included patients over 18 years old; all patients who had been treated by intra-arterial mechanical thrombectomy with thrombus were retrieved for histopathological analysis, and LAO patients who did not have visible thrombi for analysis were excluded from this study. Data regarding patient demographics, clinical presentation, treatment strategies, outcome, imaging findings, and stroke pathogenesis were collected. The stroke subtypes were classified using the Trial of Org 10172 in Acute Stroke Treatment classifications (12). Drinking as defined more than 50 ml per day. Long-term bedridden was defined as staying in bed for more than 3 months.

(2) Valvular heart disease (VHD) patients with a left atrial thrombus removed during cardiac valvectomy or replacement were enrolled retrospectively at the same time. The inclusion criteria were age >18 years old, patients who underwent valvular surgery for valve lesions such as mitral stenosis or aortic stenosis, and patients who had thrombus material for histopathological analysis.

(3) Patients who underwent carotid endarterectomy (CEA) and had carotid atherosclerotic plaque specimens were included retrospectively at the same time. Patients with carotid atherosclerotic plaque stenosis over 75% and >18 years of age were included.

### Thrombus Sample Staining and Histopathological Analysis

Atrial thrombi were obtained during cardiac surgery, carotid atherosclerotic plaques were obtained from carotid endarterectomy, and emboli were retrieved during mechanical thrombectomy (stent-retriever and contact aspiration). All samples were flushed with 0.9% saline, gently placed on sterile gauze wipes, and then fixed in 10% buffer formalin for 2–8 h. The volume of the formaldehyde was approximately 10 times the size of the thrombus. After the sections were treated with solvents and embedded in paraffin, the thrombus organization was identified in the largest section of 6 serial sections that were each 4 μm; these sections were stained with hematoxylin and eosin. Another section was immunohistochemically stained for CD31 (mouse monoclonal, 1:100; Dako Denmark A/S, ready to use) by stepwise procedures. All thrombus slices were dried in a 60°C oven for 30 min, dewaxed in xylene and rehydrated in decreasing ethanol grades. A 6% solution of hydrogen peroxide in water and the biotin-blocking reagent were used to block endogenous peroxidase and endogenous biotin successively. Primary antibodies against CD31 were added and incubated for 22 min at normal temperature, followed by streptavidin-peroxidase conjugation and incubation for 22 min. The sections were counterstained with hematoxylin, dehydrated in increasing grades of ethanol, cleared in xylene and mounted.

**Figure 1:**
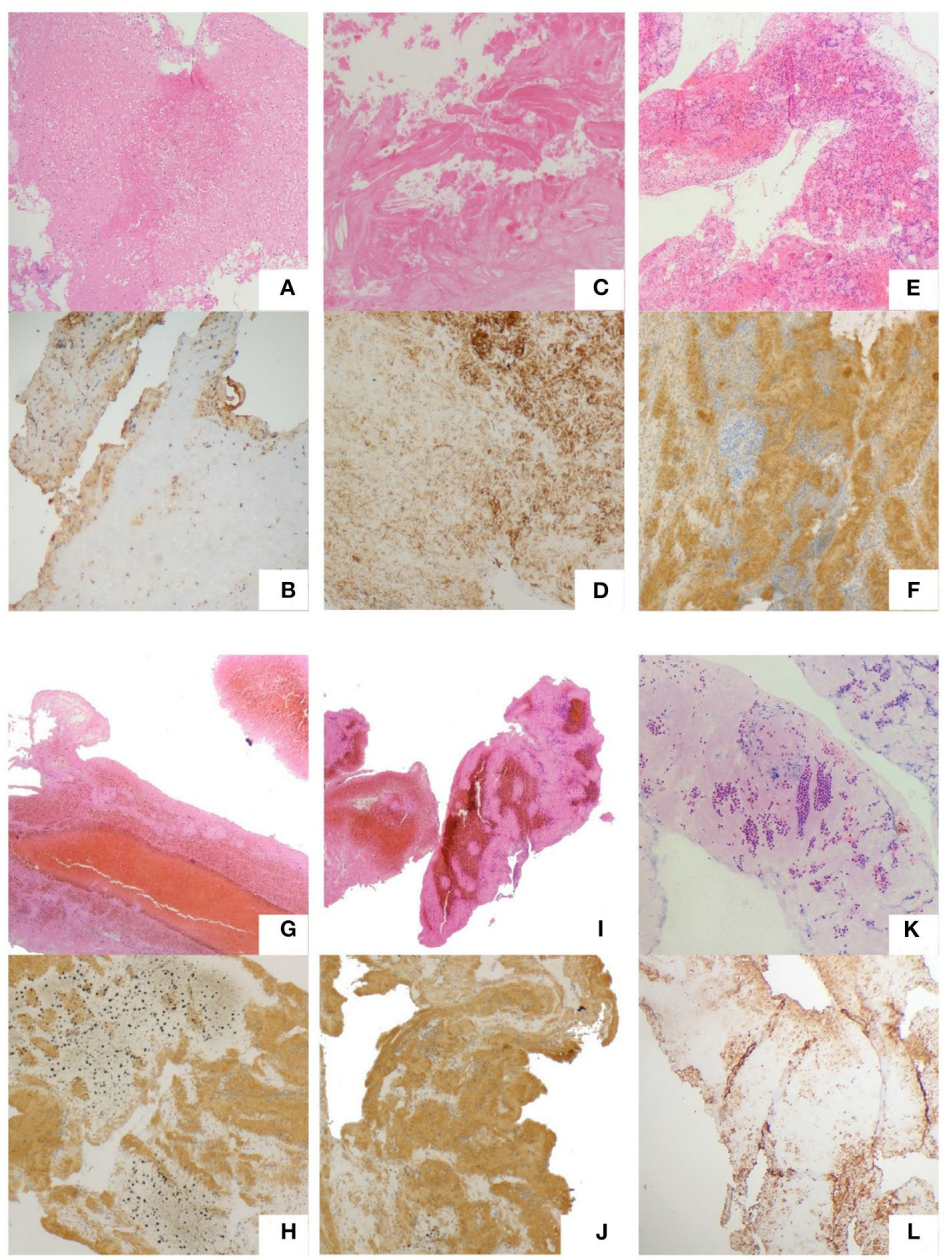
Typical thrombi and plaques. Atrial thrombi of VHD patients. **(A,B)** Carotid atherosclerotic plaques of CEA patients. **(C,D)** Thrombi of CE. **(E,F)** LAA. **(G,H)** SOE. **(I,J)** SUE. **(K,L)** Hematoxylin and eosin staining. **(A,C,E,G,I,K)** Red color represents red blood cells; pink regions represent fibrin; and blue dots represent WBCs; CD31 immunostaining. **(B,D,F,H,J,L)** Dark brown represents platelets (magnification, 200⋄).

For the H&E staining images, red represents red blood cells; pink regions represent fibrin; and blue dots represent WBCs. For CD31 immunohistochemical staining, dark brown represents platelets, and other components are not colored (Figure 1). Histological quantification was performed using ImageJ 1.52a (Image Processing and Analysis in Java; National Institutes of Health, USA) as per the standard operating procedure. The mean value of each clot component was calculated. All observers were blinded to the study groups. The calculation formulas are as follows:

$$\begin{array}{l} Target\;area\;proportion\;\left(\%\right)=measurement\;target\;area\\ \;(fibrin/platelet/red\;blood\;cells)/\\ \;-\left(the\;total\;areof\;pictures\;the\;blank\;area\right){\;}^{*}100\%\\ \text{The number of WBCs}=\text{WBC count}/\\ \;\left(the\;total\;area\;of\;pictures-the\;blank\;area\right){\;}^{*}10^{6}. \end{array}$$

### Statistical Analysis

The data were assessed using ANOVA or the Kruskal-Wallis method, depending on the type of data. The correlation analysis between basic information about the participants and the structure of thrombosis was calculated by Spearman's method. All the analyses above were performed by SPSS (Version 23).

## Results

### Patient Characteristics

There were 88 patients from whom thrombi were retrieved during mechanical thrombectomy; 175 with no thrombus were excluded from the study. The reasons of no thrombus retrieval including partial recanalization before thrombectomy, arterial sclerosis, too small thrombi, thrombi escaped to a distal blood vessel or thrombectomy failed. All patients in group VHD (*n* = 12) had valvular heart disease, and nine patients had atrial fibrillation. All patients in the CEA group (*n* = 11) had no atrial fibrillation. There were statistically noticeable differences among the VHD, CEA, and LAO groups in the proportion of patients with atherosclerotic coronary heart disease and atrial fibrillation. There were significantly more VHD patients with atherosclerotic coronary heart disease and atrial fibrillation in the VHD group than in the other two groups (Table 1). The cause of stroke was determined by two stroke neurologists based on all clinical information and imaging available for each patient. LAO patients were divided into these subtypes: (1) CE (83.3% of patients with atrial fibrillation, *n* = 46), (2) large artery atherosclerosis (LAA, *n* = 25), and (3) stroke of other determined etiology (SOE, *n* = 6), including patients with arterial dissection, (4) stroke of undetermined etiology (SUE, *n*= 11), (5) small-artery occlusion (SAO, *n* = 0). LAA is defined as artery-to-artery embolism in our study, excluding *in-situ* atherothrombosis. Among the LAO subtypes, the proportion of coronary atherosclerotic heart disease and atrial fibrillation in the CE group was significantly higher than that in the other three groups. The distribution of the National Institutes of Health Stroke Scale (NIHSS) score and Alberta Stroke Program Early CT Score (ASPECTS) before thrombectomy and the modified Rankin Scale (mRS) at 3 months after discharge were the same in each group. There was no significant difference among the subtypes (Table 2).

**Table 1:** Clinical patient characteristics.

	**LAO**	**VHD**	**CEA**	***P***
	**(*n* = 88)**	**(*n* = 12)**	**(*n* = 11)**	
Age (years)	63.36 ± 15.97	60.25 ± 9.69	65.00 ± 4.69	0.71
Male	59 (67.05%)	7 (58.33%)	6 (54.55%)	0.63
Risk factors				
Hypertension	46 (52.27%)	1 (8.33%)	5 (45.45%)	0.39
Diabetes mellitus	38 (43.18%)	3 (25%)	5 (45.45%)	0.47
Coronary artery disease	29 (32.95%)	12 (100%)	4 (36.36%)	<0.001
Dyslipidemia	24 (27.27%)	3 (25%)	1 (9.09%)	0.42
Smoking	24 (27.27%)	1 (8.33%)	4 (36.36%)	0.27
Drinking	11 (12.5%)	0	3 (27.27%)	0.14
Atrial fibrillation	38 (43.18%)	9 (75%)	0	<0.001
History of stroke or TIA	18 (20.45%)	5 (41.67%)	2 (18.18%)	0.24
Long-term bedridden	4 (4.55%)	1 (8.33%)	0	0.63

**Table 2:** Clinical characteristics of stroke subtypes (TOAST).

	**LAA**	**CE**	**SOE**	**SUE**	***P***
	**(*n* = 25)**	**(*n* = 46)**	**(*n* = 6)**	**(*n* = 11)**	
Age (years)	64.19 ± 15.06	65.04 ± 14.43	55.80 ± 14.17	58.20 ± 21.70	0.37
Male	14 (56.00%)	24 (52.17%)	3 (50.00%)	7 (63.64%)	0.91
Risk factors					
Hypertension	15 (60.00%)	21 (45.65%)	1 (16.67%)	3 (27.27%)	0.43
Diabetes mellitus	12 (48.00%)	16 (34.78%)	1 (16.67%)	6 (54.55%)	0.32
Coronary artery disease	2 (8.00%)	21 (45.65%)	1 (16.67%)	3 (27.27%)	<0.001
Dyslipidemia	10 (40.00%)	9 (19.57%)	1 (16.67%)	3 (27.27%)	0.28
Smoking	7 (28.00%)	10 (21.74%)	1 (16.67%)	3 (27.27%)	0.90
Drinking	6 (24.00%)	3 (6.52%)	0	1 (9.09%)	0.12
Atrial fibrillation	2 (8.00%)	33 (71.74%)	0	0	<0.001
History of stroke or TIA	6 (24.00%)	10 (21.74%)	0	1 (9.09%)	0.44
Long-term bedridden	0	4 (8.70%)	0	0	0.28
Intravenous thrombolysis	5 (20.00%)	13 (28.26%)	1 (16.67%)	4 (36.36%)	0.69
Times of thrombectomy	2 ± 0.83	2 ± 2.00	2 ± 0.71	3 ± 2.81	0.41
Baseline NIHSS score	17 ± 8.65	16 ± 5.98	14 ± 6.20	18 ± 9.38	0.69
ASPECT score	9 ± 0.89	9 ± 2.12	8 ± 1.48	9 ± 1.25	0.60
mRS 0-2	10 (40.00%)	27 (58.70%)	4 (66.67%)	2 (18.18%)	0.06

ANOVA was used for data conforming to a normal distribution, and the Kruskal-Wallis method was used for data not conforming to a normal distribution.

### Histological Analysis

Stained slices were scanned by using an Olympus BX43 microscope and digital camera to take panoramic photos (magnification, 200◇). The quantification of fibrin, red blood cells and platelets was used to measure the area covered by various components in the image [%], and the WBCs were used to automatically measure the particle number of the threshold set (Table 3).

**Table 3:** Differences in the components among the six groups.

	Fibrin (%)^*^	Red blood cell (%)^*^	White blood cell^*^	Platelet (%)^*^
LAA (*n* = 25)	22.96 (17.81, 28.11)	53.44 (49.91, 56.97)	171.91 (120.01, 223.79)	23.48 (17.69, 29.27)
CE (*n* = 46)	35.91 (31.44, 40.39)	35.70 (32.04, 39.36)	198.47 (166.82, 230.12)	28.43 (22.68, 34.19)
SOE (*n* = 6)	26.33 (12.31, 40.36)	41.83 (25.95, 57.71)	158.92 (73.92, 243.94)	31.83 (20.82, 42.85)
SUE (*n* = 11)	39.73 (27.97, 51.49)	38.18 (31.01, 45.35)	155.24 (86.85, 223.64)	22.09 (10.69, 33.49)
VHD (*n* = 12)	40.83 (34.70, 46.97)	37.08 (31.70, 42.47)	107.38 (48.82, 165.93)	22.08 (16.00, 28.16)
CEA (*n* = 11)	19.91 (17.81, 28.11)	59.00 (47.93, 70.07)	206.11 (169.82, 242.41)	21.09 (7.94, 34.24)
*P*	0.000	0.000	0.127	0.513

* *The data are presented as the median percentage (interquartile range, IQR)*.

#### Differences in the Composition of Thrombi Among VHD, CEA, and LAO Patients

VHD thrombi showed the highest percentage of fibrin, the lowest percentage of red blood cells and the lowest WBC count. The percentage of CEA was the lowest, and CEA thrombi showed the highest numbers of red blood cells and WBCs. The percentage of LAO was between them and showed statistically noticeable differences.

#### Comparison of Stroke Subtypes and VHD and CEA

**Figure 2:**
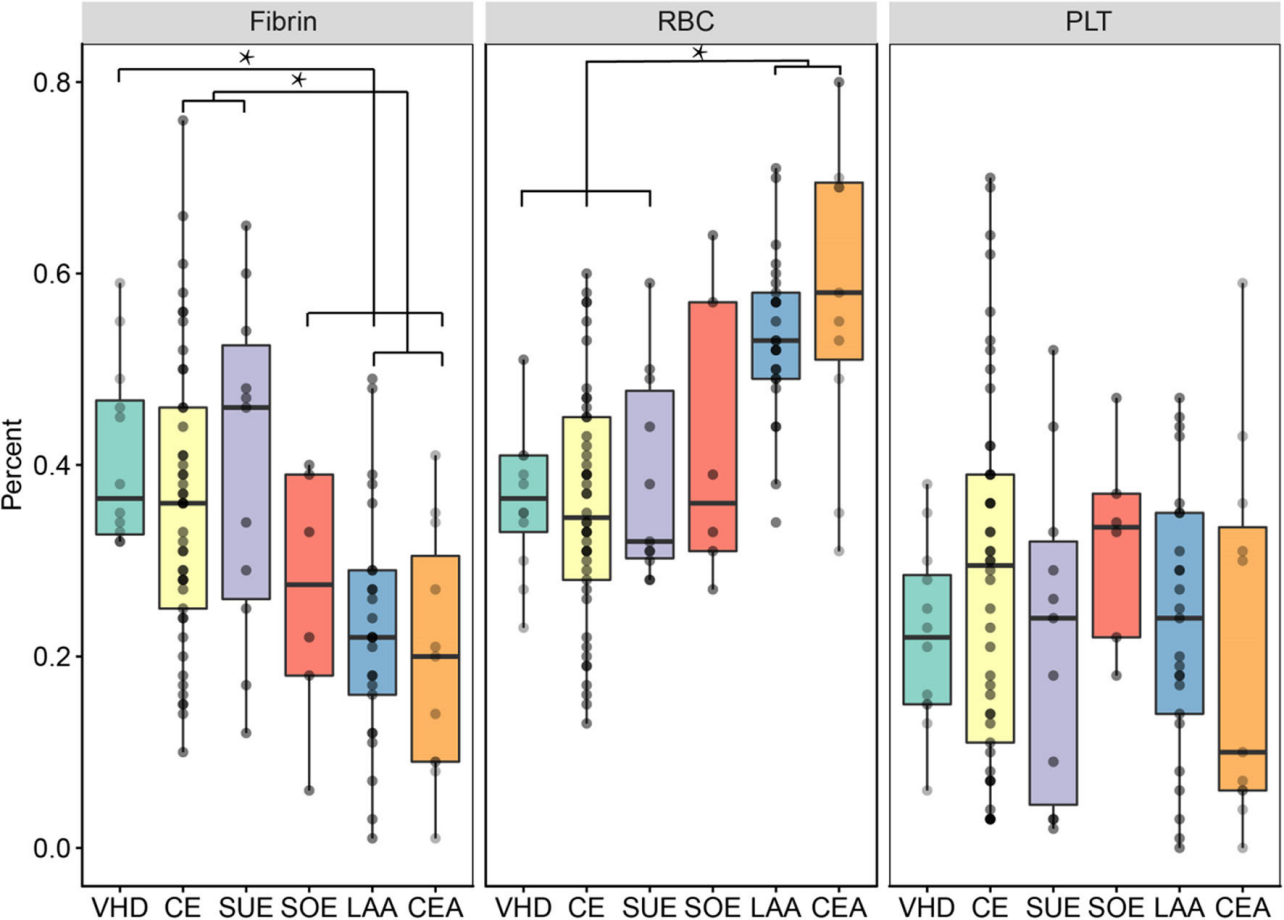
Main sample components of VHD, CEA, and subtypes of TOAST. RBCs, red blood cells; PLT, platelets; VHD, valvular heart disease; CE, cardioembolic; SUE, stroke of undetermined etiology; SOE, stroke of other determined etiology; LAA, large artery atherosclerosis; CEA, carotid endarterectomy. *P < 0.05.

**Figure 3:**
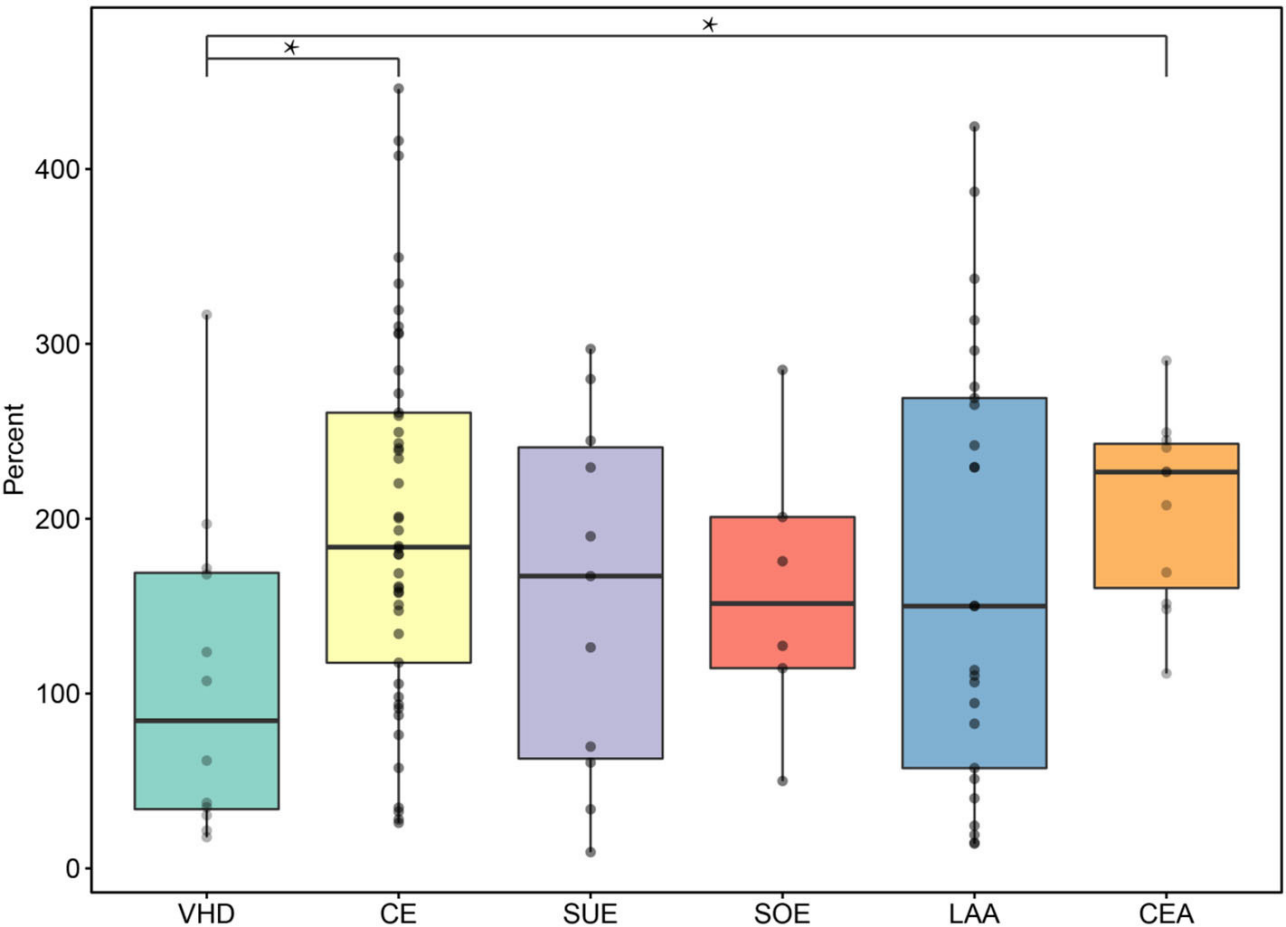
WBC counts of VHD, CEA, and subtypes of TOAST. VHD, valvular heart disease; CE, cardioembolic; SUE, stroke of undetermined etiology; SOE, stroke of other determined etiology; LAA, large artery atherosclerosis; CEA, carotid endarterectomy. *P < 0.05.

The percentages of fibrin in thrombi of the CE and SUE subtypes were higher than those in thrombi of the LAA and SOE subtypes, showing statistically noticeable differences between other stroke subtypes. LAA thrombi showed the highest red blood cell count among stroke subtypes but less fibrin (Figure 2). Thrombi of the CE subtype had more WBCs than those of the other stroke subtypes. CEA thrombi had significantly more WBCs than those of the VHD subtype (Figure 3). No statistically noticeable differences in platelets between groups were found.

## Discussion

In this study, we analyzed the histological characteristics of thrombi from patients with acute stroke of LAO and compared the histological composition with atrial thrombi and carotid atherosclerotic plaques for the first time, which showed a stronger reference. The sample size in the present study was larger than those in many previous studies.

In our cases, there were significantly more VHD patients with atherosclerotic coronary heart disease and atrial fibrillation than in the other two groups, suggesting that coronary heart disease and atrial fibrillation are both important risk factors for cardiac thrombosis. The age of the patients, sex, hypertension, diabetes, hyperlipidemia, smoking, alcohol consumption, history of stroke or transient ischemic attack, and long-term bedridden status were not different among the LAO, VHD, and CEA groups. All included patients with different stroke subtypes had no significant differences in baseline data, preoperative NIHSS scores, ASPECT scores or mRS scores 3 days after the operation. The proportion of patients with CE thrombi with coronary atherosclerotic heart disease and atrial fibrillation was significantly higher than that among the other three subtypes, which was related to the TOAST classification criteria.

It is worth noting that thrombus composition plays a crucial role in recanalization therapy in LAO patients (13). We found that the percentage of fibrin in the VHD group was significantly higher than that in the other groups, and the contents of red blood cells and WBCs were lower. Previous studies have suggested that atrial thrombi are composed of extensive fibrin and a small number of embedded red blood cells and platelets (14). A study that examined the histopathology of atrial thrombus extraction during cardiac valve surgery in patients with atrial fibrillation and thrombus of cardiac embolization origin from the iliofemoral artery and the subclavian brachial artery during vascular surgery found that the fibrin area of most atrial thrombi was significantly larger than the platelet area (15). The pathogenesis of atrial thrombosis is multifactorial and is mainly related to blood stasis of the left atrium with poor contractility, followed by the hypercoagulable state of blood (16). Especially in mitral stenosis or atrial fibrillation, abnormal stagnation of blood flow in the left atrium and left atrial appendage is important for left atrial thrombosis (17–20). The larger the atrial thrombus is, the less likely it is to dissolve because the denser fibrin network in the thrombus is more resistant to endogenous thrombolysis (21). Boeckh-Behrens et al. (8) found that patients with cardiogenic embolism had a significantly higher proportion of fibrin/platelets in their thrombi than in thrombi from other stroke subtypes, similar to our findings. The fibrin in the CE group was as dominant as that in the VHD group and was significantly more abundant than that in the other stroke subtypes and the CEA group. The fibrin content of both CE and SUE thrombi was close to that of atrial thrombi. Yuki et al. (22) recently found in pig models that the histological characteristics of thrombus had an effect on the process and success of mechanical thrombectomy; in particular, high fibrin content increased the difficulty of thrombectomy and decreased the success rate of mechanical thrombectomy.

We found that the level of WBCs in the CE thrombus of LAO was significantly higher than that in the VHD group and higher than that in the other subtypes of acute LAO. Boeckh-Behrens et al. studied the histopathology of 34 patients with acute anterior circulative stroke and found that the WBC content of CE thrombi was significantly higher than that in thrombi of other causes of acute stroke (23), which was consistent with our findings. WBCs can improve the overall stability of thrombi with the help of newly discovered neutrophil extracellular traps (NETs) and thus enhance resistance to thrombolysis (24–30). In terms of thrombus composition, fibrin/platelet-rich thrombi have increased friction with the vessel wall, and the reduced fusion of this thrombus with the thrombus cutter makes these thrombi more resistant to mechanical thrombectomy and more difficult to remove (31, 32). Structurally, fibrin/platelet-rich thrombi include dense fibrin/vWF structures and WBCs. Large fibrin bundles can increase the hardness of the thrombus and affect the friction coefficient and physical compression level of the thrombus (5). NETs of WBCs can also change the structure of fibrin to make it more resistant to mechanical force (32–34). These thrombotic characteristics make it difficult for CE thrombi to achieve good results through thrombolysis or mechanical thrombectomy.

Our findings suggested that thrombi in the CEA group had the highest proportion of red blood cells, followed by those in the LAA group, whose red blood cell content was higher than that in thrombi of the other LAO subtypes. Niesten et al. (35) studied thrombi after mechanical thrombectomy in 22 patients with acute stroke. They found that thrombi originating from LAA had the highest proportion of red blood cells, while there was no statistically significant difference in the proportion of fibrin and platelets among the other stroke subtypes. A limitation of the study was the small number of patients. Red blood cells play a major role in the transition from stable atherosclerotic lesions to unstable lesions, leading to occlusion (8, 23, 32, 34). Therefore, the proportion of red blood cells originating from unstable atherosclerotic plaques is higher, especially in cerebral thrombi, and most LAA thrombi are acute thromboembolic events that occur after intraplaque hemorrhage (36–38). With age, the vascular wall is aging and prone to damage. After vascular endothelial cells are damaged, the production of coagulation kinases increases, and the anticoagulant prostacyclin decreases, leading to the formation of thrombi, which contain more red blood cells (6, 39). A study of 649 patients who underwent mechanical thrombectomy for AIS concluded that mechanical thrombectomy may be more effective in the treatment of LAA stroke than CE stroke (40). Many studies have shown that compared with CE stroke, stroke thrombi rich in red blood cells are easier to obtain through intravascular treatment and easier to recanalize through rt-PA intravenous thrombolysis (41, 42).

Our study found that the plaques of the CEA group were rich in WBCs. Many studies have shown that inflammation plays many key roles in the development and progression of atherosclerosis (43, 44) and that infiltration of WBCs into the carotid plaque surface may be a key step in promoting plaque formation or carotid artery occlusion (45).

Currently, the SUE thrombi showed histological characteristics and compositions similar to those of cardiogenic thrombi seen in several studies (8–10), suggesting that a cardiac origin is the main cause of undetermined etiology stroke (10). Other studies have assessed the composition of thrombi in patients with SUE and found no significant difference between these thrombi and the proportion of cardiogenic or large artery atherosclerotic thrombi (23). However, our study found that the fibrin and red blood cell contents of SUE thrombi were very close to those of CE and LAA thrombi, which supported the current findings of stroke of undetermined etiology.

The SOE group in our study included patients with arterial dissection. These thrombi were characterized by a higher proportion of red blood cells and significantly less fibrin content than atrial thrombi of VHD, suggesting that such thrombi might be emboli caused by the formation and shedding of a hematoma in the arterial wall. In a previous study of three patients with arterial dissection, thrombi of mixed but predominantly red blood cells were observed (35), and thrombectomy in this group was relatively effective. The limitation of this histological analysis of thrombi is that the sample size is small, and further study is needed.

In conclusion, the proportions of fibrin and red blood cell content of CE and SUE thrombi were the closest to those of atrial thrombi in VHD. CE thrombi originated from cardiac thrombi, suggesting that most SUE thrombi may also originate from the heart. The proportions of red blood cells and fibrin in thrombi in the LAA group were close to those in thrombi in the CEA group, suggesting that the formation of carotid atherosclerotic plaques may be related to stroke of LAA. These results provide a reference for the study of embolus sources. There were significantly more WBCs in the CE group and plaque in the CEA group than in the atrial thrombus group, suggesting that in addition to traditional thrombolysis and thrombectomy, anti-inflammatory therapy may improve the success rate of recanalization in the treatment of CE stroke. For patients with carotid artery plaque and stenosis, early anti-inflammatory therapy may have a certain inhibitory effect on plaque rupture or carotid artery occlusion, which provides a theoretical basis for clinical treatment.

A limitation of this study is that it is a preliminary and retrospective study. Second, the composition statistics of thrombi or plaques in each group were averages, which cannot exclude the existence of the same type of thrombus in different subtypes of a cerebral thrombus. For example, red thrombi were found in both the LAA and CE groups; thus, it is not simply considered that red thrombi are from strokes of the LAA or CE. The results of this study provide only a reference for the etiological source of stroke. Furthermore, intravenous thrombolysis may also affect the composition of the thrombus, but the proportion of such cases is small. However, the samples we selected were all previously treated with intravascular therapy after thrombolysis failure, and the proportion of thrombolysis was small; thus, this effect was excluded.

## Data Availability Statement

The datasets presented in this study can be found in online repositories. The names of the repository/repositories and accession number(s) can be found in the article/Supplementary Material.

## Ethics Statement

All patients who were treated in our hospital signed informed consent for medical research of their images and specimens. This study was conducted according to the recommendations of guidelines from the Institutional Review Board of the First Affiliated Hospital of Jinan University. All protocols and procedures of our research were carried out in conformity to the Helsinki Declaration.

## Author Contributions

YL and MG performed all the pathological experiments, performed data analyses, and wrote the manuscript. DL and SH performed the patients' TOAST classification. YS and JL performed data analyses. XZ, XX, and DY wrote and edited the manuscript. LH and HQ contributed to the conception and design of the study and wrote and edited the manuscript. All authors contributed to the article and approved the submitted version.

## Conflict of Interest

The authors declare that the research was conducted in the absence of any commercial or financial relationships that could be construed as a potential conflict of interest.
